# SIGMA PHI OMEGA PRESIDENTIAL SYMPOSIUM: ITS ROLE IN ADVANCING THE FUTURE OF GERONTOLOGISTS AND HEALTH PROFESSIONALS

**DOI:** 10.1093/geroni/igac059.183

**Published:** 2022-12-20

**Authors:** Diane Martin, Katarina Friberg Felsted

## Abstract

Sigma Phi Omega, The International Academic Honor and Professional Society in Gerontology (aka Sigma Phi Omega), was established in 1980 to recognize excellence of those who study gerontology and aging, and the outstanding service of professionals who work with or on behalf of older persons. The formation of this society provided a much-needed link between educators, practitioners, and administrators in various settings where older persons are served. Through the efforts of the international office and executive board officers, Sigma Phi Omega builds avenues to further their members’ academic and professional gerontological excellence. The goals of Sigma Phi Omega are achieved primarily through activities of local chapters at higher education institutions worldwide. Sigma Phi Omega chapters serve as links within their respective communities to promote interaction between gerontology educators, students, alumni, and local professionals. This international organization has a laser focus on excellence within gerontology and health professions education. In this session, the first presentation will provide the history of Sigma Phi Omega and its outreach efforts; the second presentation will focus on the future goals of Sigma Phi Omega to expand its role as an international honor society in preparing gerontologists, service providers and health professionals working with or on behalf of older adults; and the third presentation will focus on the SPO Chapters and their relationship with the piloted Gerontological Society of America Student Chapters.

